# Editorial 2020: Changing publishing and academic culture

**DOI:** 10.1098/rsos.192197

**Published:** 2020-01-15

**Authors:** Jeremy K. M. Sanders

**Affiliations:** Department of Chemistry, University of Cambridge, Cambridge CB2 1EW, UK

It is over 5 years since the launch of *Royal Society Open Science* [1], and 4 since I became its Editor-in-Chief [2]. Much has changed in that time.

In the last year alone, the journal has pioneered several innovations in the Royal Society's publishing portfolio. At the beginning of 2019, we moved from an optional open peer review model to publishing the peer review and decision history of all accepted papers [3]. I have been glad to see a growing community response to our introduction of Replication studies to support greater reproducibility across the sciences, with our first published Replications in Psychology and Cognitive Neurosciences [4].

The journal has remained at the forefront of the Royal Society's mission to support and promote high-quality science to a global public. Our publications have not only dispelled widely held views about the relationship of heavy metal music and video games to violent behaviour but also reported distressing news of the looming extinction of a porpoise species native to Mexico [5–7]. We have also published advice from David Spiegelhalter and co-workers [8] to journalists, researchers and policy makers on how to communicate uncertainty about facts, numbers and science.

Furthermore, while the world's politicians appear ever-more insular in outlook, I am reassured by the global nature of science. While the journal's administrative office is based at the Royal Society in London, an overwhelming majority of submissions received and published by the journal are from non-UK-based authors (nearly 90% and 85%, respectively). I speak for all of the journal's Editors and staff when I offer my thanks for the support of all our authors, readers and reviewers [9].

That said, my role as Editor-in-Chief, and coincidentally as Chair of the Athena Forum, which advocates for greater diversity [10], gives me an unusual perspective on some of the challenges a research journal faces.

The role of Editor-in-Chief at *Royal Society Open Science* means that I am generally invited to offer a view on the more troublesome manuscripts or matters that arise. In a recent unfortunate example, a female early career researcher whose paper had been accepted for publication received several unpleasant messages from disgruntled correspondents regarding a matter largely resolved (to the best of the journal's ability). I wanted to draw upon this example for a number of reasons. Firstly, it is sadly not uncommon for promising young scientists to experience such toxic behaviour and working environments. It is one thing to disagree with someone scientifically, it is quite another to send messages implying that an individual's career will be ruined by associating themselves with Prof. A or Dr B. Where individuals from any background, gender, ethnicity, sexuality or other protected characteristic work in an environment that feels unsupportive at best, and bullying at worst, it is no great surprise that they feel they must leave that environment. Indeed, in a separate case recently, a number of promising female post-doctoral researchers indicated to their supervisor that they would leave science because their broader scientific environment was too unpleasant to work in. When individuals are so harassed or shouted down, or simply ignored, by their ostensible colleagues that they leave active research, the community is poorer for it, and we should be ashamed when this happens.

A second reason that I wanted to touch on these cases (and others I have witnessed) is that we each have a responsibility to ensure as welcoming a working environment as possible—not just in the laboratory but throughout the wider culture of research and academia—and we can all do more to develop a more inclusive and diverse research infrastructure. *Royal Society Open Science,* for instance, is working to improve the geographical and gender representation of its Editors and reviewers. We welcome volunteers to both roles, especially from under-represented groups [11]. Thirdly, a more diverse environment is not just good for the workplace (scientifically or otherwise) but it is also a lot of fun! Some of my most rewarding experiences as a teacher, researcher and now Editor-in-Chief have been when I have engaged with a wide pool of people from very different backgrounds.

It may be uncomfortable for some readers that I am using this editorial as something of a soapbox, but I would urge you to ask yourself, ‘What am I doing now and what could I be doing to make the workplace more supportive and inclusive?' Only by working together can poor behaviour be called out and tackled, to be replaced by a better environment and, dare I say it, a better society for everyone, regardless of who you are or where you come from.

I will be encouraging the Editors, staff, authors, reviewers and readers of *Royal Society Open Science* to take up this rallying cry in 2020. I hope you will join me.

## Data accessibility

This article has no additional data.
